# Gut microbiome alterations during gastric cancer: evidence assessment of case–control studies

**DOI:** 10.3389/fmicb.2024.1406526

**Published:** 2024-05-15

**Authors:** Ruimin Zhang, Yingxin Wu, Wantao Ju, Senlin Wang, Yanjun Liu, Hongmei Zhu

**Affiliations:** ^1^Institute of Biomedical Engineering, College of Medicine, Southwest Jiaotong University, Chengdu, China; ^2^Section for Gastrointestinal Surgery, Department of General Surgery, The Third People’s Hospital of Chengdu, Aliated Hospital of Southwest Jiaotong University & The Second Aliated Hospital of Chengdu, Chongqing Medical University, Chengdu, China; ^3^School of Life Science and Engineering, Southwest Jiaotong University, Chengdu, China; ^4^Medical Research Center, The Third People’s Hospital of Chengdu, The Aliated Hospital of Southwest Jiaotong University, Chengdu, China

**Keywords:** gastric cancer, gastric microbiota, stomach, carcinogenesis, meta-analysis

## Abstract

**Objectives:**

The study aims to systematically identify the alterations in gut microbiota that observed in gastric cancer through comprehensive assessment of case–control studies.

**Methods:**

The systematic literature search of PubMed, Embase, Cochrane Library, and Web of Science was conducted to identify case–control studies that compared the microbiomes of individuals with and without gastric cancer. Quality of included studies was evaluated with the Newcastle-Ottawa Quality Assessment Scale (NOS). Meta-analyses utilized a random-effects model, and subgroup and sensitivity analyses were performed to assess study heterogeneity. All data analyses were performed using the “metan” package in Stata 17.0, and the results were described using log odds ratios (log ORs) with 95% confidence intervals (CIs).

**Results:**

A total of 33 studies involving 4,829 participants were eligible for analysis with 29 studies provided changes in α diversity and 18 studies reported β diversity. Meta-analysis showed that only the Shannon index demonstrated statistical significance for α-diversity [−5.078 (−9.470, −0.686)]. No significant differences were observed at the phylum level, while 11 bacteria at genus-level were identified significant changed, e.g., increasing in *Lactobacillus* [5.474, (0.949, 9.999)] and *Streptococcus* [5.095, (0.293, 9.897)] and decreasing in *Porphyromonas* and *Rothia* with the same [−8.602, (−11.396, −5.808)]. Sensitivity analysis indicated that the changes of 9 bacterial genus were robust. Subgroup analyses on countries revealed an increasing abundance of *Helicobacter* and *Streptococcus* in Koreans with gastric cancer, whereas those with gastric cancer from Portugal had a reduced *Neisseria*. Regarding the sample sources, the study observed an increase in *Lactobacillus* and *Bacteroides* in the gastric mucosa of people with gastric cancer, alongside *Helicobacter* and *Streptococcus*. However, the relative abundance of *Bacteroides* decreased compared to the non-gastric cancer group, which was indicated in fecal samples.

**Conclusion:**

This study identified robust changes of 9 bacterial genus in people with gastric cancer, which were country-/sample source-specific. Large-scale studies are needed to explore the mechanisms underlying these changes.

**Systematic Review:**

Unique Identifier: CRD42023437426 https://www.crd.york.ac.uk/prospero/display_record.php?ID=CRD42023437426.

## 1 Introduction

Gastric cancer, a prevalent and malignant tumor, is a major global health concern and one of the leading causes of cancer-related death (Sung et al., 2021). According to World Health Organization (2020), it ranked third in terms of cancer-related mortality worldwide. The development of gastric cancer involves multiple factors, including smoking, alcohol consumption, genetics, and alterations in the gut microbiota (Aviles-Jimenez et al., 2014; Rawla and Barsouk, 2019). The gut microbiome consists of a community of microorganisms that reside in the intestines, including bacteria, fungi, viruses, and other types of microorganisms. These communities of microbes perform crucial functions in human physiology and metabolism, including digestion and regulation of the immune system. Furthermore, they are closely linked to human health. In healthy individuals, the gut microbiota tends to remain stable. However, dysbiosis, an imbalance in the gut microbiota, can arise due to medication use, environmental changes, and dietary variation. Dysbiosis of the gut microbiota has been linked to the development of diverse ailments (Fan and Pedersen, 2021).

The relationship between gastric cancer and the gut microbiota has been a primary subject of investigation. Several studies indicate variations in the composition of the gut microbiota between gastric cancer and those without, implying a crucial role of the gut microbiota in the development of gastric cancer. However, the specific changes in bacterial composition vary between studies. Some studies suggest a decrease in microbial diversity (Coker et al., 2018; Peng et al., 2023), whereas others suggest an increase in diversity (Wang et al., 2016; Castaño-Rodríguez et al., 2017). Besides, the specific microbial species implicated in different studies also vary. For example, Castaño-Rodríguez et al. (2017) research detected an enrichment of *Lactococcus*, *Fusobacterium*, and *Veillonella* in gastric cancer compared to precancerous stages. Wei et al. (2023) found notable variations in the prevalence of *Streptococcus*, *Rhodococcus*, and *Ochrobactrum* between individuals with gastric cancer and healthy individuals. Meanwhile, Peng et al. (2023) indicated an increase in some genera such as *Lautropia* and *Lactobacillus*, and a decrease in others notably *Peptostreptococcus* and *Parvimonas* among the gastric cancer group in contrast to the control group. Additionally, the exact role of the gut microbiota in the development of gastric cancer remains the subject of ongoing debate. Although some researchers contend that alterations in the gut microbiota may be an independent risk factor for gastric cancer, others argue that it is a secondary factor. Lastly, differences in the source of samples, gene regions selected for sequencing, sequencing platforms, reference databases, and data analysis methods lead to variations in the results of different studies (Nearing et al., 2022). Thus, further research is essential to investigate the mentioned issues thoroughly. Meta-analysis is a possible method to address above issues by synthesizing published studies and combining the effects of different factors to produce more effective results.

Therefore, this study aims to fill the gaps of previous studies by meta-analysis to summarize research on changes in the gut microbiota of people with gastric cancer and without gastric cancer to elucidate microbial changes during gastric cancer development.

## 2 Materials and methods

### 2.1 Registration

The systematic review and meta-analysis was registered in the International Prospective Register of Systematic Reviews (PROSPERO) with the registration number CRD42023437426, which was reported according to the Preferred Reporting Items for Systematic Reviews and Meta-analyses (PRISMA) guidelines (Liberati et al., 2009).

### 2.2 Data sources and search strategy

A systematic search was executed utilizing computerized bibliographic databases such as PubMed, Embase, Cochrane Library, and Web of Science, covering all records up until April 4, 2023. The search strategy combined MeSH and free terms using the Boolean operators “AND” and “OR.” For instance, PubMed was searched with the following query: (microbio*[Title/Abstract]) AND (“stomach neoplasms”[MeSH Terms] OR “cancer of stomach”[Title/Abstract] OR “stomach cancers”[Title/Abstract] OR “gastric cancer*”[Title/Abstract]). The detailed search protocols for each scientific database are shown in Supplementary Table S1.

### 2.3 Inclusion and exclusion criteria

The inclusion criteria were as follows: (1) adult diagnosed with gastric cancer through gastroscopic biopsy; (2) the control group consisted of non-gastric cancer individuals undergoing either endoscopy or biopsy, including healthy individuals and those with precancerous lesions such as chronic gastritis or intestinal metaplasia; (3) reporting the changes in gut microbiota composition/diversity; and (4) case–control study.

Study was excluded if it met any of the following situations: (1) people had undergone gastric cancer-related treatments, such as surgery, chemotherapy, radiation therapy, and immunotherapy; (2) pregnant women were involved; (3) samples were from oral, skin, or oropharyngeal; and (4) changes in the gut microbiota cultured in specific media were excluded since the culture conditions exert a significant influence on microbiota data (Goodrich et al., 2014; Widder et al., 2016). Additionally, abstracts, editorials, comments, and studies written in languages other than English were also excluded.

### 2.4 Study selection and data extraction

Two researchers (Zhang and Wu) screened the searching results of databases according to the inclusion and exclusion criteria independently. Titles and abstracts were screened firstly, and then the full texts were reviewed to identify eligible studies. Four researchers (Zhang, Wu, Ju, and Wang) independently exacted the following information from each eligible study: the study ID (first author and publication year), country, sample size, age, gender, *H. pylori* infection status, sample source, method for measuring the microbiome, DNA extraction methods, annotation database, composition and diversity of the gut microbiome in people with and without gastric cancer, and the differences in the gut microbiome between the two groups. The exacted data were cross-checked by four investigators. Any disagreement during study selection and data extraction was settled by consultation with the fifth researcher (Zhu) to reach a consensus.

### 2.5 Quality assessment

The quality of the included studies was evaluated using the Newcastle-Ottawa Quality Assessment Scale (NOS) (Wells et al., 2000). The NOS consists of selection, comparability, and measurement of exposure factors. Each study can receive a maximum of nine points. Two researchers (Zhang and Ju) assessed each study independently, and any discrepancies were resolved through consensus or with the assistance of a third researcher (Zhu) if necessary.

### 2.6 Data synthesis

Meta-analysis was conducted using the “metan” package in Stata 17.0 with a random-effects model, and heterogeneity was assessed using the *I*^2^ statistic. Based on the alterations in the diversity and abundance of microbiota between people with and without gastric cancer, these results were transposed into a binary format to indicate whether there was an increase. The results of meta-analyses were presented as log odds ratios (log OR) and their 95% confidence intervals (CI). A log OR significantly less than 0 indicated a decrease in the abundance of a certain microbial community in people with gastric cancer compared to those without gastric cancer, while a log OR significantly greater than 0 indicated an increase in the abundance of a certain microbial community in people with gastric cancer. For a more intuitive evaluation, Forest plots were utilized. Meta-regression and subgroup analyses were performed to investigate potential heterogeneity, considering the country, sample source, amplification region of the 16S rRNA gene, and microbial database. Sensitivity analysis was performed on studies with a sample size exceeding 50. Funnel plot, Egger’s and Begg’s test were conducted to detect potential publication bias which was corrected by trim-and-fill analysis (Mavridis and Salanti, 2014). All *p*-values were two-tailed, and those *p* < 0.05 were considered statistically significant.

## 3 Results

### 3.1 Literature search and studies overview

**Figure 1:**
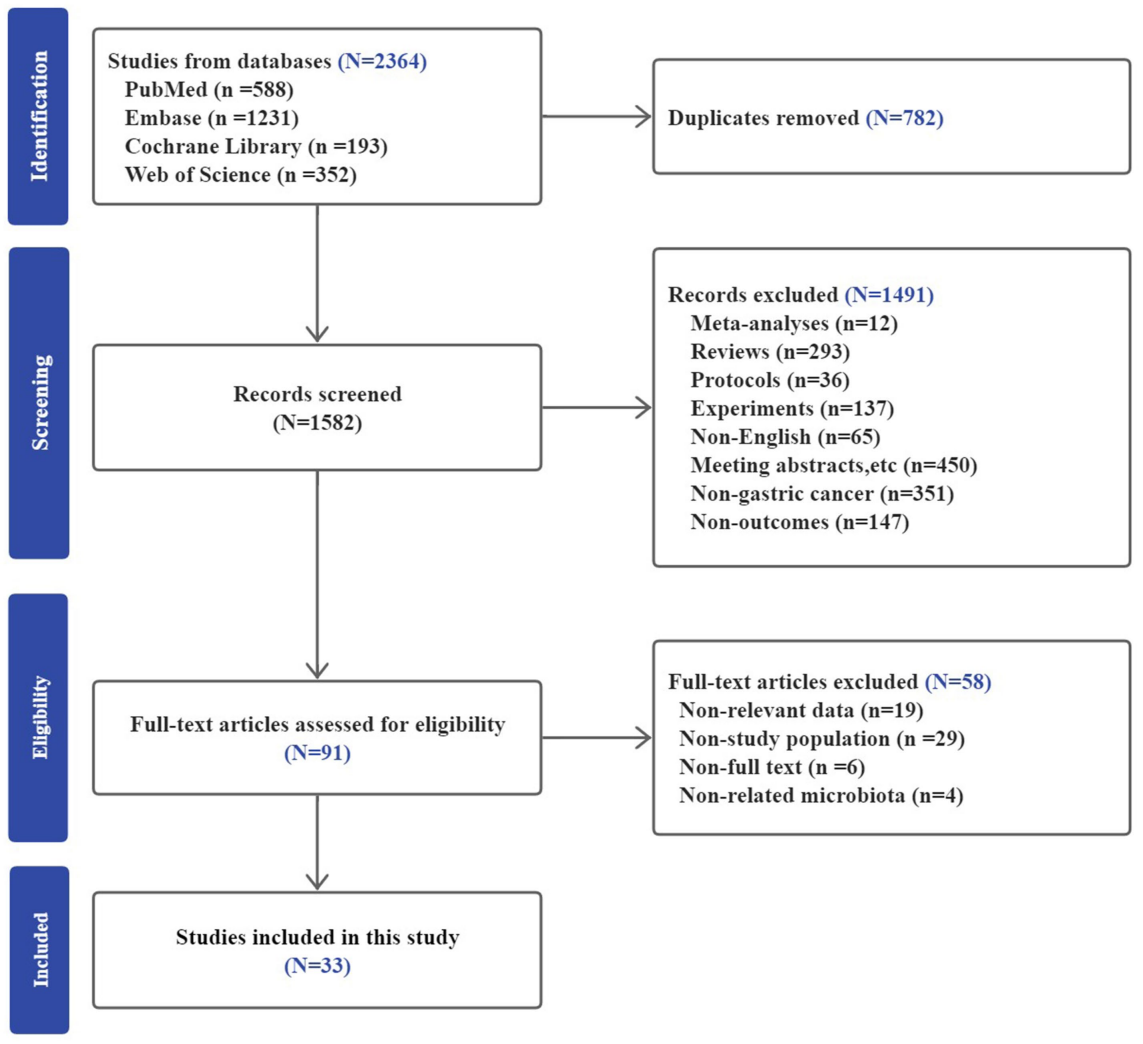
Description of the selection of the included studies.

A total of 2,364 studies were identified from PubMed, Embase, Cochrane Library, and Web of Science. After duplicates removal, 1,582 studies remained for screening the title and abstract. Out of the 1,582 studies, 1,491 studies were excluded. The excused studies included meta-analyses, reviews, protocols, meeting abstracts, experiments and non-English articles, and those that did not focus on gastric cancer or provide the required results. As a result, 91 articles entered the full-text review stage. Finally, 33 studies met the eligible criteria and were included in the meta-analysis. The selection process is illustrated in Figure 1.

Table 1 shows the main characteristics of the included studies which published between 2014 and 2023. The majority of studies were conducted in Asian countries, including China (*n* = 21), Korea (*n* = 8), and Mongolia (*n* = 1). Three studies were conducted in Europe, two in Portugal and one in Lithuania. In addition, one study was conducted in several countries. A total of 4,829 participants were included in these studies, with males outnumbering females. Fourteen studies reported on people infected with *Helicobacter pylori*. A total of 25 studies collected samples from gastric mucosal biopsies during gastroscopy, while four studies used fecal samples (Liang et al., 2019; Qi et al., 2019; He et al., 2022; Kim et al., 2022), and four studies used gastric juice samples (Park et al., 2022; Sun et al., 2022; Peng et al., 2023; Wei et al., 2023). Twenty-seven studies used 16S gene sequencing technology, but with different amplified regions. Of these, one study amplified the V1–V2 (Nikitina et al., 2023), V1–V4 (Wei et al., 2023), V1–V8 (Pimentel-Nunes et al., 2021), V4–V5 (Chen et al., 2019), V5 (Eun et al., 2014), and V5–V6 (Ferreira et al., 2018) regions, respectively. Two studies amplified V1–V3 (Jo et al., 2016; Sohn et al., 2017) and six studies amplified V4 (Coker et al., 2018; Wang et al., 2020; He et al., 2022; Li et al., 2022; Miao et al., 2022; Peng et al., 2023). The most commonly amplified region was V3–V4, with thirteen studies using this region. Three studies did not specify the region amplified (Wang et al., 2016; Castaño-Rodríguez et al., 2017; Deng et al., 2021; Wu et al., 2021). To study the fungal composition of the gut microbiota, one study used the ITS2 region for PCR amplification (Yang et al., 2022). Nine studies did not report the specific gene sequence database used, while the remaining studies mainly relied on databases such as SILVA (*n* = 9), Greengenes (*n* = 7), NCBI (*n* = 6), Ezbio (*n* = 2), EzTaxon-e (*n* = 2), and RDP (*n* = 1).

**Table 1:** Characteristics of the included studies.

Study ID	Country	Age	Sample size (male/female)	*H. pylori* status	Sample source	DNA extraction method (region amplified) database used	NOS
Eun et al. (2014)	Korea	Chronic gastritis: 50.4 ± 11.5 IM: 57.5 ± 7.3 GC: 65.7 ± 11.3	Chronic gastritis:10 (4/6) IM: 10 (7/3) GC: 11 (6/5)	Chronic gastritis: 70% IM: 40% GC: 64%	Gastric mucosal biopsies	Phenol/chloroform method and a DNA clean-up kit; 16S rRNA(V5); SILVA database	8
Jo et al. (2016)	Korea	GC: Hp (−), 61.8 ± 10.92; Hp (+), 54.1 ± 12.50; Control: Hp (−), 62.3 ± 13.61; Hp (+), 55.9 ± 10.97	GC: 34 (24/10) Control: 29 (12/17)	GC: 44.1% Control: 55.1%	Gastric mucosal biopsies	iNtRON Biotechnology; 16S rRNA(V1–V3); EzTaxon-e database	8
Wang et al. (2016)	China	55.8 ± 13.5	Chronic gastritis: 212 GC: 103 (200/115)	Chronic gastritis: 45.3% GC: 49.5%	Gastric mucosal biopsies	Qiagen Dneasy blood and tissue kit; 16S rRNA(region NR); Ribosomal database	7
Castaño-Rodríguez et al. (2017)	China	GC: 62.08 GU: 64.75 FD: 49.55	GC: 12 (5/7) GU: 4 (4/0) FD: 20 (13/7)	GC: 91.7% GU: 100% FD: 50%	Antral gastric biopsies	Isolate II RNA mini kit and Tetro cDNA synthesis kit; 16S rRNA (region NR); –	6
Li et al. (2017)	China	Normal: 49.13 Gastritis: 48 IM: 53.22 GC: 53.43 Eradication: 52.18	Normal: 8 (3/5) Gastritis: 9 (2/7) IM: 18 (8/10) GC: 14 (10/4) Eradication: 11 (3/8)	Normal: 0% Gastritis: 100% IM: 66.7% GC: 28.6% Eradication: 9%	Antrum and corpus gastric biopsies	QIAGEN DNeasy Kit; 16S rDNA(V3–V4); Greengene database	8
Sohn et al. (2017)	Korea	GC: Hp (−), 68; Hp (+): 52.8 Control: Hp (−), 53.5; Hp (+), 55.67	GC: Hp (−), 2; Hp (+),5; (3/4) Control: Hp (−), 2; Hp (+), 3; (2/3)	GC: 28.57% Control: 40%	Gastric mucosal (antrum and body) biopsies	iNtRON Biotechnology Kit; 16S rRNA(V1–V3); EzTaxon-e database	6
Yu et al. (2017)	China Mexico	Non-malignant: China, 60.8; Mexico, 64.5; Tumor, NA	non-malignant: China, 77; Mexico, 80. (27/130) tumor: China, 80; Mexico, 54. (62/72)	–	Tumor tissue and matched non-malignant tissue	Allprep RNA/DNA/Protein mini kit (QIAGEN) and QIAamp DNA mini kit (QIAGEN); 16S rRNA(V3–V4); Greengenes and BioProject database	8
Coker et al. (2018)	China	–	AG: 77 SG: 74 IM: 17 GC: 39	Xi’an: 50.8–53.9% Mongolia:44.8–47.5%	Antrum, body and fundus for SG, AG and IM. Biopsies cancer lesions and adjacent non-cancerous tissues of GC.	QIAamp DNA Mini Kit; 16S rRNA(V4); SIL VA database	7
Ferreira et al. (2018)	Portugal	Chronic gastritis: 43.6 ± 7.0 GC: 58.8 ± 13.2	Chronic gastritis:81 (79/2); GC: 54 (32/22)	–	Gastric biopsies or surgical specimens of non-neo plastic gastric mucosa adjacent to the tumour	– 16S rRNA(V5–V6); Greengenes database	7
Hsieh et al. (2018)	China	Gastritis: 32.2 IM: 46.3 GC: 68.6	Gastritis:9 (3/6) IM: 7 (4/3) GC: 11 (5/6)	Gastritis: 55.5% IM: 85.7% GC: 0	Gastric biopsies	TRI Reagent^®^; 16S rRNA(V3–V4); NCBI database	7
Chen et al. (2019)	China	60	(*N* = 62, gastric adenocarcinoma) 124 gastric tissue samples (cancerous and paired non-cancerous tissues)	29%	Subtotal gastrectomy	Lysozyme, proteinase K and SDS, phenol chloroform isoamyl alcohol, glycogen, sodium acetate and cold isopropanol; 16S rRNA(V4–V5); NCBI and SIL VA database	5
Gunathilake et al. (2019)	Korea	Control: 51.53 ± 7.21 GC: 53.68 ± 9.60	Control: 288 (181/107) GC: 268 (172/96)	Control: 93.4% GC: 99.6%	Gastric mucosa biopsy	MagAttract DNA Blood M48 kit; 16S rRNA(V3–V4); NCBI database	7
Liang et al. (2019)	China	GC: 52.3 ± 11.2 HC: 53.4	GC: 20 HC: 22	–	Fecal samples	E.Z.N.A Stool DNA Kit; – –	8
Qi et al. (2019)	China	GC: 58.06 ± 11.24 HC: 45.58 ± 8.86	GC: 116 (96/20) HC: 88 (53/35)	–	Fecal samples	E.Z.N.A.^®^ Stool DNA Kit; 16 S rDNA(V3–V4); NCBI and SIL VA database	9
Gantuya et al. (2020)	Mongolian	46.4	GC: 48 Normal: 22 Gastritis: 20 Atrophy: 66 IM: 40 (59/137)	–	Gastric mucosal biopsies	DNeasy Blood & Tissue Kit; 16 S rRNA(V3–V4); SIL VA database	7
Gunathilake et al. (2020)	Korea	Control: 51.53 ± 7.21 GC: 53.68 ± 9.60	Control:288 GC:268 (353/203)	Control: 93.4% GC:99.63%	Gastric mucosal biopsy	MagAttract DNA Blood M48 kit; 16 S rRNA(V3–V4); Ezbio database	9
Wang et al. (2020)	China	HC: 45.63 CG: 49.04 IM: 56.93 IN: 62.16 GC:57.35	HC: 30 (15/15) CG: 21 (10/11) IM: 27 (16/11) IN: 25 (18/7) GC:29 (18/11)	HC: 0% CG: 28.57% IM: 29.63% IN: 60% GC:58.62%	Gastric mucosal biopsy	QIAamp DNA Mini Kit; 16S rRNA(V4); Greengenes database	7
Deng et al. (2021)	China	Chronic superficial Gastritis: 45–70 GC: 46–75	Chronic superficial gastritis: 25 (13/12) GC: 34 (24/10)	Chronic superficial gastritis: 0% GC: 26.47%	Chronic superficial gastritis: the antrum (*n* = 10), c o r p u s (*n* = 7) and cardia (*n* = 8) GC: cancer in the antrum (*n* = 19) and corpus (*n* = 15).	– 16S rRNA(region NR); RDP and NCBI database	9
Gunathilake et al. (2021)	Korea	–	GC: 268 HC: 288 (353/203)	–	Gastric mucosal biopsies	MagAttract DNA Blood M48 kit; 16 S rRNA(V3–V4); Ezbio database	8
Kadeerhan et al. (2021)	China	Normal/SG: 53.8 ± 7.8 CAG: 53.4 ± 9.3 IM: 58.5 ± 7.7 DYS/GC: 57.6 ± 6.4	Normal/SG: 35 (13/22) CAG: 52 (29/23) IM: 67 (43/24) DYS/GC: 25 (20/5)	Normal/SG: 74.3% CAG: 92.3% IM: 94.0% DYS/GC: 60.0%	Gastric mucosal biopsies	QIAamp DNA Mini Kit; 16S rRNA(V3–V4); Greengenes and SIL VA database	7
Pimentel-Nunes et al. (2021)	Portugal	Controls: 53 (27–82) Extensive atrophy/metaplasia: 63 (53–87) Early gastric cancer: 70 (43–89)	Controls: 17 (11/6) Extensive atrophy/metaplasia: 12 (5/7) Early gastric cancer: 31 (17/14)	Controls: 41% Extensive atrophy/metaplasia: 25% Early gastric cancer: 13%	Biopsy fragment from the antrum and the corpus	NZY Tissue gDNA isolation kit; 16S rRNA(V1–V8); –	7
Wu et al. (2021)	China	GC: 62.50 ± 6.64 SG: 61.78 ± 6.25	GC: 18 (15/3) SG: 32 (24/8)	–	Gastric mucosa biopsy samples, samples were collected from the greater curvature of the antrum, the lesser curvature of the antrum, the greater curvature of the stomach body, the lesser curvature of the stomach body, and the fundus.	E.Z.N.A.^®^ Stool DNA Kit; 16S rRNA(region NR); SIL VA database	7
Zhang et al. (2021)	China	SG: 56.00 ± 10.25 AG: 63.58 ± 6.69 GIN: 64.80 ± 9.93 GC: 69.87 ± 11.57	SG: 17 AG: 10 GIN: 5 GC: 15 (20/27)	–	Gastric mucosal biopsies	E.Z.N.A ^®^Stool DNA Kit; 16S rRNA(V3–V4); SILVA database	8
He et al. (2022)	China	–	Gastric cancer: 30 Healthy people: 30	–	Fecal samples	CTAB method; 16S rDNA(V4); –	7
Kim et al. (2022)	Korea	GC: 62.9 ± 10.2 Control: 50.7 ± 13.6	GC: 45 (31/14) CG:49 IM:43 (Control:47/45)	0%	Histological evaluation using endoscopic biopsy tissues.	DNeasy PowerSoil Kit; 16S rRNA(V3–V4); NCBI and taxonomy databases	7
Li et al. (2022)	China	GC: 63.5 HC: 55	GI Cancer: 130 (93/37) HC: 147 (84/63)	–	Fecal samples	NucleoSpin Soil DNA Kit; 16S rRNA(V4); Greengenes database	6
Miao et al. (2022)	China	SG: 47.40 ± 12.37 AG: 45.77 ± 13.62 GMAH: 64.00 ± 11.83 GC: 69.60 ± 6.91	SG: 15 (7/8) AG: 13 (8/5) GMAH: 8 (5/3) GC: 15 (11/4)	SG: 26.7% AG: 61.5% GMAH: 100% GC: 73.3%	Gastric mucosal biopsies	QIAamp PowerFecal Pro DNA Kit; 16S rRNA(V4); Greengenes database	7
Park et al. (2022)	Korea	Gastritis: 59.8 ± 12.5 Gastric adenoma: 65.3 ± 9.6 EGC: 62.7 ± 10.8 AGC: 58.8 ± 15.8	Gastritis: 16 (6/10) Gastric adenoma: 16 (12/4) EGC: 36 (25/11) AGC: 20 (14/6)	–	Gastric juice	DNeasy PowerSoil kit; 16S rRNA(V3–V4); SILVA database	8
Sun et al. (2022)	China	SG: 50.29 ± 14.31 AG: 60.67 ± 10.71 IM: 60.27 ± 14.89 Dys: 62.71 ± 12.21 GC: 71.67 ± 9.87	SG: 56 (27/29) AG: 9 (5/4) IM: 27 (12/15) Dys: 29 (15/14) GC: 13 (7/6)	0%	Gastric mucosal biopsies and Gastric juice	E.Z.N.A^®^ Soil DNA Kit; 16S rRNA(V3–V4); –	7
Yang et al. (2022)	China	GC: 60.59 ± 12.73 HC: 52.64 ± 10.92	GC: 22 (16/6) HC: 11 (4/7)	–	Gastric mucosal biopsies	E.Z.N.A. R soil DNA Kit; ITS2 rRNA PCR; –	6
Nikitina et al. (2023)	Lithuania	–	GC: 76 HC: 29	–	Gastric mucosal biopsies	AllPrep DNA/RNA Mini kit; 16S rRNA(V1–V2); –	8
Peng et al. (2023)	China	HC: 49.5 (32–60) GPL: 48.5 (32–59) GC: 59.5 (44–81)	HC: 22 (13/9) GPL: 22 (10/12) GC: 16 (10/6)	HC: 27.3% GPL: 40.9% GC: 68.8%	Gastric juice	QIAamp^®^ FAST DNA Stool Mini Kit; 16S rRNA(V4); –	8
Wei et al. (2023)	China	Healthy: 51.61 ± 11.68 GC: 67.97 ± 9.24	Healthy: 61 (37/24) GC: 78 (58/20)	–	Gastric juice	– 16S rDNA(V1–V4); –	7

IM, intestinal metaplasia; HP, *Helicobacter pylori*; GU, gastric ulcers; SG, superficial gastritis; AG, atrophic gastritis; AGC, advanced gastric cancer; CAG, chronic atrophic gastritis; CG, chronic gastritis; CSG, chronic superficial gastritis; DYS, dysplasia; EGC, early gastric cancer; F, female; FD, functional dyspepsia; F/M, female to male ratio; GA, gastritis; GC, gastric cancer; GAD, gastric adenoma; GI, gastrointestinal; GIN, gastric intraepithelial neoplasia; GMAH, gastric mucosal atypical hyperplasia; GPL, gastric precancerous lesions; HC, healthy control; IN, intraepithelial neoplasia; M, male; NCBI, National Center for Biotechnology Information; NOS, Newcastle-Ottawa Scale; RDP, ribosomal database project; SIL VA, small subunit rRNA database.

The NOS was used to assess the quality of the included studies. Three studies scored nine points, 10 studies scored eight points, 15 studies scored seven points, and the remaining studies scored six points or less. The detailed quality assessment scores can be found in the Supplementary Table S2.

### 3.2 Primary outcomes

#### 3.2.1 Biodiversity

Out of the 33 studies analyzed, 29 focused on investigating the α-diversity of the gastrointestinal microbiota and 18 studies explicitly reported differences in *β*-diversity between people with and without gastric cancer (refer to Supplementary Table S3). However, due to the diverse use of different indices and variations in expression across studies, quantitative analysis of *β*-diversity was not available. Meta-analysis showed that only the Shannon index demonstrated statistical significance for *α*-diversity [−5.078 (−9.470, −0.686)] (Supplementary Figure S1).

#### 3.2.2 Differences in the microbial composition

Eighteen studies with five phylum-level gut microbiotas were available for meta-analysis: *Actinobacteria*, *Bacteroidetes*, *Firmicutes*, *Clostridia*, and *Proteobacteria*. No statistically significant differences between people with and without gastric cancer in terms of these five phylum-level gut microbiotas were identified by meta-analysis. Supplementary Table S4 presents the changes in relative abundance at the phylum level for people with and without gastric cancer in individual studies.

A total of 30 studies reported data on the relative abundance of bacteria at genus-level in people with gastric cancer compared to those without it (Supplementary Table S5). The most frequently reported genera in gastric cancer patient samples were *Lactobacillus* and *Helicobacter*. Meta-analysis of the data from these studies indicated significant changes in the abundance of 11 out of 32 evaluated genera, with nine exhibiting an increase and two exhibiting a decrease. The increased abundance of genera such as *Lactobacillus* and *Streptococcus* was characterized by log odds ratio (95% CI) of 5.474 (0.949, 9.999) and 5.095 (0.293, 9.897), respectively. In contrast, *Porphyromonas* and *Rothia* exhibited a significant and identical decrease in people with gastric cancer, with −8.602 (−11.396, −5.808). Table 2 presents a detailed summary of the findings.

**Table 2:** Meta-analysis of changes on genus level between gastric and non-gastric cancer patients.

Genus	No. of studies	Simple size	Log odds ratio (95% CI)	*p*-value	*I*^2^
*Lactobacillus*	11	1027	5.474 (0.949, 9.999)	0.020	93.60%
*Streptococcus*	11	969	5.095 (0.293, 9.897)	0.038	93.80%
*Achromobacter*	2	184	8.716 (5.923, 11.510)	1.995e-09	0.00%
*Bacillus*	2	303	9.661 (6.876, 12.446)	1.058e-10	0.00%
*Capnocytophaga*	2	226	8.643 (5.847, 11.439)	1.995e-09	0.00%
*Clostridium*	3	204	7.994 (5.706, 10.283)	7.553e-12	0.00%
*Dialister*	2	257	8.995 (6.204, 11.787)	1.350e-09	0.00%
*Klebsiella*	2	253	9.141 (6.203, 12.080)	1.995e-09	9.70%
*Slackia*	2	254	8.909 (6.116, 11.703)	1.362e-09	0.00%
*Porphyromonas*	2	182	−8.602 (−11.396, −5.808)	1.995e-09	0.00%
*Rothia*	2	182	−8.602 (−11.396, −5.808)	1.995e-09	0.00%

### 3.3 Subgroup analyses

Subgroup analyses in gastric cancer microbiome research revealed significant findings, highlighting the impact of methodological and geographical variables (Table 3). At the phylum level, *Actinobacteria* exhibited significant abundance changes across different 16S rRNA regions, with a pronounced increase in the V1–V3 region (6.748, 95% CI, 3.608, 9.889) and a decrease in the V4 region when annotated with Greengenes (−10.334, 95% CI: −13.116, −7.552). Subgroup analysis on geographical regions found a higher prevalence of *Helicobacter* and *Streptococcus* in the Korean population, with 9.936 (4.611, 15.261) and 5.651 (2.795, 8.508), respectively, at genus-level. In contrast, the Portuguese population exhibited a reduced prevalence of *Neisseria* with −9.006 (−11.795, −6.218). The prevalence of *Lactobacillus* varied across different 16S rRNA gene amplification regions, with 8.365 (5.567, 11.162) for the V4 region and 7.449 (4.642, 10.257) for the NR region. In gastric biopsy samples, *Lactobacillus* was less prevalent with a log OR of −5.939 (0.300, 11.578), while *Bacteroides* showed a higher abundance, evidenced by a log OR of 11.154 (8.227, 14.082). The analysis of gastric acid samples showed a higher prevalence of *Helicobacter* and *Streptococcus*, with 8.552 (5.757, 11.348) and 8.598 (5.803, 11.393), respectively. Those results suggested that the bacteria were country-/sample source-specific. Database analysis revealed a notable increase in the prevalence of *Lactobacillus* among individuals diagnosed with gastric cancer in studies utilizing Greengenes, with a mean of 9.598 (6.813, 12.383). Conversely, studies referencing NCBI indicated an increase in *Fusobacterium*, with a mean of 8.163 (5.200, 11.127).

**Table 3:** Statistically significant bacterial groups identified by the meta-analysis in subgroup analysis.

Outcome	Subgroup	Bacterial groups	*N*	Sample size	Log odds ratio	*p*-value	*I*^2^
					(95% CI)		
Phylum	**Method**						
	16S rRNA(V1–V3)	*Actinobacteria*	2	75	6.748 (3.608, 9.889)	2.54E-05	18.50%
	16S rRNA(V4)	*Bacteroidetes*	2	409	10.334 (7.552, 13.116)	6.62E-13	97.20%
		*Actinobacteria*	2	409	−10.334 (−13.116, −7.552)	6.62E-13	0.00%
	**Database**						
	EzTaxon-e	*Actinobacteria*	2	75	6.748 (3.608, 9.889)	2.54E-05	18.50%
	Greengenes	*Proteobacteria*	3	469	9.531 (7.254, 11.808)	1.87E-15	0.00%
		*Actinobacteria*	2	409	−10.334 (−13.116, −7.552)	6.62E-13	0.00%
	NCBI and SILVA	*Actinobacteria*	2	328	10.143 (7.362, 12.923)	1.17E-12	0.00%
		*Firmicutes*	2	328	−10.143(−12.923, −7.362)	1.17E-12	0.00%
Genus	**Country**						
	Korea	*Helicobacter*	2	592	9.936 (4.611, 15.261)	2.78E-04	72.50%
		*Streptococcus*	2	33	5.651 (2.795, 8.508)	1.27E-04	0.00%
	Portugal	Neisseria	2	195	−9.006 (−11.795, −6.218)	7.33E-10	0.00%
	**Method**						
	16S rRNA (V4)	*Lactobacillus*	2	170	8.365 (5.567, 11.162)	7.90E-09	0.00%
	16S rRNA (region NR)	*Lactobacillus*	2	86	7.449 (4.642, 10.257)	2.64E-07	0.00%
	**Sample source**						
	Stomach	*Bacteroides*	12	680	11.154 (8.227, 14.082)	4.88E-13	9.90%
		*Lactobacillus*	8	718	5.939 (0.300, 11.578)	0.039	93.80%
	Gastric juice	*Helicobacter*	2	175	8.552 (5.757, 11.348)	4.04E-09	0.00%
	Feces	*Streptococcus*	2	177	8.598 (5.803, 11.393)	3.95E-09	0.00%
		*Bacteroides*	3	306	−8.800 (−11.079, −6.520)	4.66E-13	0.00%
	**Database**						
	Greengenes	*Lactobacillus*	2	267	9.598 (6.813, 12.383)	5.69E-11	0.00%
	NCBI	*Fusobacterium*	2	151	8.163 (5.200, 11.127)	1.01E-07	10.60%

*N*, the number of studies.

### 3.4 Meta-regression

Meta-regression analysis aimed to identify sources of heterogeneity in gastric cancer microbiome studies. Results showed that geographic differences significantly affect *Bacteroidetes* and *Firmicutes* abundance (Supplementary Table S6). Specifically, the analysis indicated a strong negative association of *Bacteroidetes* with country (−21.91816, *p* < 0.001) and a positive association for *Firmicutes* (9.307176, *p* = 0.018). Methodological factors, such as the choice of 16S rRNA gene amplification regions and databases for annotation, significantly impacted the abundance of *Actinobacteria*. The method used showed a negative coefficient (−20.59842, *p* = 0.009), while the database used showed a positive coefficient (18.17374, *p* = 0.008). Additionally, sample sources were found to contribute to the heterogeneity of Firmicutes (−19.25102, *p* = 0.006).

### 3.5 Sensitivity analysis and publication bias

After excluding studies with a sample size of less than 50, sensitivity analysis revealed trends in changes to microbial diversity indices as well as microbial community structure at the genus and phylum classification levels (Supplementary Table S7). As a result, changes in 9 out of the 11 bacterial genera identified by overall analysis were found to be robust. Notably, the analysis of the genus *Clostridium* showed a slight increase in the log OR from 7.994 to 8.227, and the *p*-value changed from an extremely low 7.55E-12 to 1.80E-07 when small-sample studies were excluded. Although the result remained statistically significant, the increase in heterogeneity to 17.70% suggested some inconsistency between studies. Regarding the Shannon index of *α*-diversity, the log odds ratio slightly decreased after exclusion, while the *p*-value rose from 0.023 to 0.048. This implied that the negative association’s statistical significance was somewhat strengthened. Overall, excluding small-sample studies caused only limited changes in the log odds ratios and *p*-values.

The funnel plots indicated possible publication bias in the meta-analysis of microbial diversity and abundance related to gastric cancer. Asymmetries were observed for several bacteria. The funnel plot for Shannon appears symmetrical, indicating minimal bias, which was supported by a non-significant Egger’s test. However, a significant Begg’s test for Shannon suggested that further scrutiny might be necessary. For *Actinobacteria*, both Egger’s and Begg’s tests showed a low probability of bias. The plot for *Proteobacteria* displayed slight asymmetry, but only the trim-and-fill method indicated the need for adjustment, adding three studies to the left. *Helicobacter*, *Lactobacillus*, and *Streptococcus* exhibited asymmetrical plots. Begg’s test suggested potential bias for the latter two, although Egger’s test results did not align with this for all (Supplementary Table S8).

## 4 Discussion

This meta-analysis aggregated data from 33 studies and explored the evolution of the gut microbiome from pre-cancerous conditions to the development of gastric cancer. In comparison to previous studies, our analysis was more comprehensive. Initially, we conducted a meta-analysis, followed by subgroup analysis, sensitivity analysis, and meta-regression. Additionally, we conducted a detailed analysis of publication bias. According to our study, a pattern of reduced microbial diversity was found, which is consistent with earlier studies (Liu et al., 2022; Li et al., 2023). However, it is important to note that earlier studies may have been limited by the scope of their sample selection, potentially not capturing the full spectrum of microbiome variability associated with gastric cancer. Our analysis builds upon and expands these findings by incorporating a broader and more diverse datasets, enhancing the comprehensiveness and generalizability of our conclusions. The reduction in microbial diversity observed in various studies emphasizes its potential impact on the immune system’s ability to respond to cancer. This highlights the critical role of the gut microbiome in the progression of gastric cancer.

The Shannon index, often used to measure species richness and evenness (Lozupone and Knight, 2008), was significantly reduced in people with gastric cancer compared to pre-cancerous conditions in this study, which implies a decrease in the diversity of the gastric microbial ecosystem when gastric cancer develops. The reasons for the decrease are not yet clear. A previous study suggested that it might be a result of factors such as gastric acid and *Helicobacter pylori* infection reshaping the microbial community during the carcinogenic process (Wang et al., 2018). However, changes in diet, use of antibiotics or other medications (David et al., 2014; Altveş et al., 2020) were reported to be associated with the reduction in Shannon index.

At the genus level, an increase in *Lactobacillus* and *Streptococcus*, alongside a decrease in *Rothia* and *Porphyromonas*, were identified in this meta-analysis. The variation in bacterial abundance is thought to influence the immune system’s ability to detect and eliminate cancer cells. For example, an increase in *Lactobacillus* correlates with higher counts of CD3^+^ T cells (Qi et al., 2019), suggesting a complex relationship between microbiome composition and immune function. Furthermore, experimental evidence from studies such as Lertpiriyapong et al. (2014) highlighted how specific bacterial presences could trigger inflammation and promote cancer development. Interestingly, interventions such as post-surgical supplementation with *Clostridium butyricum* have been shown to modulate immune responses favorably, indicating potential therapeutic pathways (Cao et al., 2022). Epidemiological studies have suggested a correlation between the occurrence of gastric cancer and periodontal disease (Lo et al., 2021). *Porphyromonas* is one of the pathogens that cause periodontal disease (Darveau, 2010). Experiments have shown that lipopolysaccharide (LPS) from *Porphyromonas* can damage the gastric mucosal barrier, which is considered a promoting factor for cancer-related gastritis. Furthermore, LPS from *Porphyromonas* can regulate the host’s immune response (Oriuchi et al., 2024). Although direct research linking *Rothia* with gastric cancer is limited, it is important to note that *Rothia* is part of the core microbiota in the stomachs of healthy individuals (Nardone and Compare, 2015). The gut microbiota can produce butyrate, a short-chain fatty acid that has been shown to suppress the expression of PD-L1 and IL-10 in immune cells and demonstrate tumor growth inhibition potential in mouse models (Lee et al., 2024). It is speculated that *Rothia* may influence the progression of gastric cancer through its metabolic products. Future studies will likely focus on elucidating the specific mechanisms of these associations.

The roles of *Lactobacillus* and *Streptococcus* in gastric cancer are nuanced, with *Lactobacillus* associated with both anti-inflammatory effects and cancer progression, potentially serving as a biomarker for the disease (Bali et al., 2021). Similarly, *Streptococcus* adheres to gastric mucosa, influencing cancer development through metabolic and immune modulation (Spiegelhauer et al., 2020). Notably, *Streptococcus anginosus* has been implicated in exacerbating gastric inflammation and cancer progression (Fu et al., 2024). *Helicobacter pylori*’s role in gastric cancer development is significant (Plottel and Blaser, 2011). The involvement of *Helicobacter pylori*, a well-documented factor in gastric cancer, showed variability in our analysis, contrasting with findings by Liu et al. (2022), which could be attributed to methodological and sample size differences.

Due to the high heterogeneity observed in certain microbial communities in the overall analysis, a series of analyses including subgroup analysis, sensitivity analysis, and meta-regression were conducted to identify the sources of heterogeneity. Subgroup analyses revealed regional variations in bacterial communities, suggesting that dietary or environmental factors contributed to a higher prevalence of *Streptococcus* in Asian populations compared to Europeans. Geographic differences had a significant impact on the levels of *Bacteroidetes* and *Firmicutes*. Research showed that there were significant geographic differences in the composition of the gut microbiota between populations from the United States, Chile, South Africa, Kuwait and Malaysia, particularly in the distribution of *Bacteroidetes* and *Firmicutes*. In samples from the United States, *Firmicutes* dominate, followed by other regions such as South Africa. In Chilean samples, however, *Bacteroidetes* took the lead. Moreover, by calculating the ratio of *Firmicutes* to *Bacteroidetes* (F:B), it was found that the F:B in US samples was the highest, reaching 4.15, while the F:B in Chilean samples was the lowest (Kumar and Bhadury, 2023). Other studies in the Asian population also showed that geographic differences significantly affect the abundance of *Bacteroidetes* and *Firmicutes* in the gut. These differences were related to the unique dietary habits, cultural customs and environmental conditions of each region (Lim et al., 2021; Taha et al., 2023).

Additionally, methodological choices and sample sources introduced variability in the detection of bacteria such as *Bacteroides* and *Lactobacillus*. It became clear that methodological differences, including the choice of sample sources and DNA sequencing techniques, were the main cause of inconsistencies in microbiota research (Hiergeist et al., 2016; Tang et al., 2020). The choice between using feces or endoscopic biopsies as samples significantly affected the outcomes (Jalanka et al., 2015; Tropini et al., 2018), highlighting the nuanced impact of sample origin on research findings. Furthermore, variations in DNA isolation and sequencing methodologies, as well as the choice of database platforms, posed challenges in accurately differentiating microbial communities. These methodological considerations were crucial in microbiome studies, emphasizing the need for a rigorous and standardized approach to mitigate inconsistencies and enhance the comparability of results across studies. This comprehensive approach ensured that the complexities of microbial ecosystems were accurately interpreted, fostering advancements in our understanding of the microbiome’s role in health and disease. Variations in DNA isolation and sequencing methodologies, as well as database platforms, could introduce errors in microbial differentiation. Moreover, meta-regression also confirmed that geographic, methodological, and sample origin differences were the sources of heterogeneity.

Sensitivity analysis on studies with sample size no less than 50 revealed an increase in the *p*-values for *Lactobacillus* and *Streptococcus*. This change suggested that smaller studies might have influenced the results due to their high variability or specific biases. These small-sample studies sometimes showed a more significant association because of greater statistical variation or because of selective reporting and publication bias. Because most study samples were collected during health examinations, it was difficult to collect a large number of samples, which limited the ability to conduct large-scale research. Therefore, future studies should aim to expand the research scale and include a broader population to explore the potential association between these bacteria and gastric cancer.

This meta-analysis presented a detailed examination of the changes in the gut microbiome that are linked to the development of gastric cancer. It highlighted the intricate relationship between microbial diversity and cancer, the potential of microbiome-focused therapies, and the need for methodological rigor in future research. The limitations of this study, including lack of data, potential bias, and inability to include all relevant factors, highlight the need for large-scale studies. These limitations underscore the need for large-scale studies to confirm these findings and further explore the role of the microbiome in gastric cancer. Specifically, future research should focus on conducting long-term cohort studies to explore the dynamic changes in the gut microbiome during the development and progression of gastric cancer. In parallel, pathogenic mechanism studies should be conducted to understand how specific microbes promote or influence the development of gastric cancer. In addition, interventional studies could be conducted to evaluate the efficacy of specific microbes in the prevention and treatment of gastric cancer. Through these studies, we can gain a more complete understanding of the role of the microbiome in gastric cancer and provide guidance for targeted prevention and treatment strategies in the clinical setting.

## 5 Conclusion

This study identified robust changes of nine bacterial genus in people with gastric cancer, which were country-/sample source-specific, with lower *α*-diversity observed in individuals with gastric cancer. Large-scale studies are needed to explore the mechanisms underlying these changes.

## Data availability statement

The original contributions presented in the study are included in the article/Supplementary material, further inquiries can be directed to the corresponding authors.

## Author contributions

RZ: Data curation, Formal analysis, Writing – original draft, Writing – review & editing. YW: Data curation, Writing – original draft, Writing – review & editing. WJ: Data curation, Writing – review & editing. SW: Data curation, Writing – review & editing. YL: Conceptualization, Writing – review & editing. HZ: Conceptualization, Writing – review & editing.

## References

[ref1] Altveş S. Yildiz H. K. Vural H. C. (2020). Interaction of the microbiota with the human body in health and diseases. Biosci. Microbiota Food Health 39, 23–32. doi: 10.12938/bmfh.19-023, PMID: 32328397 PMC7162693

[ref2] Aviles-Jimenez F. Vazquez-Jimenez F. Medrano-Guzman R. Mantilla A. Torres J. (2014). Stomach microbiota composition varies between patients with non-atrophic gastritis and patients with intestinal type of gastric cancer. Sci. Rep. 4:4202. doi: 10.1038/srep04202, PMID: 24569566 PMC3935187

[ref3] Bali P. Coker J. Lozano-Pope I. Zengler K. Obonyo M. (2021). Microbiome signatures in a fast- and slow-progressing gastric cancer murine model and their contribution to gastric carcinogenesis. Microorganisms 9:189. doi: 10.3390/microorganisms9010189, PMID: 33477306 PMC7829848

[ref4] Cao W. Zheng C. Xu X. Jin R. Huang F. Shi M. . (2022). *Clostridium butyricum* potentially improves inflammation and immunity through alteration of the microbiota and metabolism of gastric cancer patients after gastrectomy. Front. Immunol. 13:1076245. doi: 10.3389/fimmu.2022.1076245, PMID: 36466862 PMC9714544

[ref5] Castaño-Rodríguez N. Goh K. L. Fock K. M. Mitchell H. M. Kaakoush N. O. (2017). Dysbiosis of the microbiome in gastric carcinogenesis. Sci. Rep. 7:15957. doi: 10.1038/s41598-017-16289-2, PMID: 29162924 PMC5698432

[ref6] Chen X. H. Wang A. Chu A. N. Gong Y. H. Yuan Y. (2019). Mucosa-associated microbiota in gastric cancer tissues compared with non-cancer tissues. Front. Microbiol. 10:1261. doi: 10.3389/fmicb.2019.01261, PMID: 31231345 PMC6560205

[ref7] Coker O. O. Dai Z. Nie Y. Zhao G. Cao L. Nakatsu G. . (2018). Mucosal microbiome dysbiosis in gastric carcinogenesis. Gut 67, 1024–1032. doi: 10.1136/gutjnl-2017-314281, PMID: 28765474 PMC5969346

[ref8] Darveau R. P. (2010). Periodontitis: a polymicrobial disruption of host homeostasis. Nat. Rev. Microbiol. 8, 481–490. doi: 10.1038/nrmicro2337, PMID: 20514045

[ref9] David L. A. Maurice C. F. Carmody R. N. Gootenberg D. B. Button J. E. Wolfe B. E. . (2014). Diet rapidly and reproducibly alters the human gut microbiome. Nature 505, 559–563. doi: 10.1038/nature12820, PMID: 24336217 PMC3957428

[ref10] Deng Y. Ding X. Song Q. Zhao G. Han L. Ding B. . (2021). Alterations in mucosa-associated microbiota in the stomach of patients with gastric cancer. Cell. Oncol. 44, 701–714. doi: 10.1007/s13402-021-00596-y, PMID: 33770413 PMC8213677

[ref11] Eun C. S. Kim B. K. Han D. S. Kim S. Y. Kim K. M. Choi B. Y. . (2014). Differences in gastric mucosal microbiota profiling in patients with chronic gastritis, intestinal metaplasia, and gastric cancer using pyrosequencing methods. Helicobacter 19, 407–416. doi: 10.1111/hel.12145 25052961

[ref12] Fan Y. Pedersen O. (2021). Gut microbiota in human metabolic health and disease. Nat. Rev. Microbiol. 19, 55–71. doi: 10.1038/s41579-020-0433-9 32887946

[ref13] Ferreira R. M. Pereira-Marques J. Pinto-Ribeiro I. Costa J. L. Carneiro F. Machado J. C. . (2018). Gastric microbial community profiling reveals a dysbiotic cancer-associated microbiota. Gut 67, 226–236. doi: 10.1136/gutjnl-2017-314205, PMID: 29102920 PMC5868293

[ref14] Fu K. Cheung A. H. K. Wong C. C. Liu W. Zhou Y. Wang F. . (2024). *Streptococcus anginosus* promotes gastric inflammation, atrophy, and tumorigenesis in mice. Cell 187, 882–896.e17. doi: 10.1016/j.cell.2024.01.004, PMID: 38295787

[ref15] Gantuya B. el Serag H. B. Matsumoto T. Ajami N. J. Uchida T. Oyuntsetseg K. . (2020). Gastric mucosal microbiota in a Mongolian population with gastric cancer and precursor conditions. Aliment. Pharmacol. Ther. 51, 770–780. doi: 10.1111/apt.15675, PMID: 32133670 PMC8761497

[ref16] Goodrich J. K. di Rienzi S. C. Poole A. C. Koren O. Walters W. A. Caporaso J. G. . (2014). Conducting a microbiome study. Cell 158, 250–262. doi: 10.1016/j.cell.2014.06.037, PMID: 25036628 PMC5074386

[ref17] Gunathilake M. N. Lee J. Choi I. J. Kim Y. I. Ahn Y. Park C. . (2019). Association between the relative abundance of gastric microbiota and the risk of gastric cancer: a case-control study. Sci. Rep. 9:13589. doi: 10.1038/s41598-019-50054-x, PMID: 31537876 PMC6753194

[ref18] Gunathilake M. Lee J. Choi I. J. Kim Y. I. Kim J. (2021). Association between bacteria other than Helicobacter pylori and the risk of gastric cancer. Helicobacter 26:e12836. doi: 10.1111/hel.12836, PMID: 34268831

[ref19] Gunathilake M. Lee J. Choi I. J. Kim Y. I. Yoon J. Sul W. J. . (2020). Alterations in gastric microbial communities are associated with risk of gastric cancer in a Korean population: a case-control study. Cancers 12:2619. doi: 10.3390/cancers12092619, PMID: 32937864 PMC7563352

[ref20] He F. Wang F. Yang J. Yang S. (2022). Explore and analyze the composition and characteristics of intestinal microbiota between gastric cancer patients and healthy people. Evid. Based Complement. Alternat. Med. 2022, 1–13. doi: 10.1155/2022/5834293 PMC9477631 36118097

[ref21] Hiergeist A. Reischl U. Gessner A. (2016). Multicenter quality assessment of 16S ribosomal DNA-sequencing for microbiome analyses reveals high inter-center variability. Int. J. Med. Microbiol. 306, 334–342. doi: 10.1016/j.ijmm.2016.03.005, PMID: 27052158

[ref22] Hsieh Y. Y. Tung S. Y. Pan H. Y. Yen C. W. Xu H. W. Lin Y. J. . (2018). Increased abundance of Clostridium and Fusobacterium in gastric microbiota of patients with gastric cancer in Taiwan. Sci. Rep. 8:158. doi: 10.1038/s41598-017-18596-0 29317709 PMC5760541

[ref23] Jalanka J. Salonen A. Salojärvi J. Ritari J. Immonen O. Marciani L. . (2015). Effects of bowel cleansing on the intestinal microbiota. Gut 64, 1562–1568. doi: 10.1136/gutjnl-2014-307240, PMID: 25527456

[ref24] Jo H. J. Kim J. Kim N. Park J. H. Nam R. H. Seok Y. J. . (2016). Analysis of gastric microbiota by pyrosequencing: minor role of bacteria other than *Helicobacter pylori* in the gastric carcinogenesis. Helicobacter 21, 364–374. doi: 10.1111/hel.12293, PMID: 26915731

[ref25] Kadeerhan G. Gerhard M. Gao J. J. Mejías-Luque R. Zhang L. Vieth M. . (2021). Microbiota alteration at different stages in gastric lesion progression: a population-based study in Linqu, China. Am. J. Cancer Res. 11, 561–575, PMID: 33575087 PMC7868750

[ref26] Kim H. N. Kim M. J. Jacobs J. P. Yang H. J. (2022). Altered gastric microbiota and inflammatory cytokine responses in patients with *Helicobacter pylori*-negative gastric cancer. Nutrients 14:4981. doi: 10.3390/nu14234981, PMID: 36501012 PMC9740132

[ref27] Kumar G. Bhadury P. (2023). Exploring the influences of geographical variation on sequence signatures in the human gut microbiome. J. Genet. 102:102. doi: 10.1007/s12041-023-01448-4 38073168

[ref28] Lee S. Y. Jhun J. Woo J. S. Lee K. H. Hwang S. H. Moon J. . (2024). Gut microbiome-derived butyrate inhibits the immunosuppressive factors PD-L1 and IL-10 in tumor-associated macrophages in gastric cancer. Gut Microbes 16:2300846. doi: 10.1080/19490976.2023.2300846, PMID: 38197259 PMC10793689

[ref29] Lertpiriyapong K. Whary M. T. Muthupalani S. Lofgren J. L. Gamazon E. R. Feng Y. . (2014). Gastric colonisation with a restricted commensal microbiota replicates the promotion of neoplastic lesions by diverse intestinal microbiota in the *Helicobacter pylori* INS-GAS mouse model of gastric carcinogenesis. Gut 63, 54–63. doi: 10.1136/gutjnl-2013-305178, PMID: 23812323 PMC4023484

[ref30] Li N. Bai C. Zhao L. Ge Y. Li X. (2022). Characterization of the fecal microbiota in gastrointestinal cancer patients and healthy people. Clin. Transl. Oncol. 24, 1134–1147. doi: 10.1007/s12094-021-02754-y, PMID: 35167015

[ref31] Li Y. Hu Y. Zhan X. Song Y. Xu M. Wang S. . (2023). Meta-analysis reveals *Helicobacter pylori* mutual exclusivity and reproducible gastric microbiome alterations during gastric carcinoma progression. Gut Microbes 15:2197835. doi: 10.1080/19490976.2023.2197835, PMID: 37020297 PMC10078126

[ref32] Li T. H. Qin Y. Sham P. C. Lau K. S. Chu K. M. Leung W. K. (2017). Alterations in gastric microbiota after *H. pylori* eradication and in different histological stages of gastric carcinogenesis. Sci. Rep. 7:44935. doi: 10.1038/srep44935, PMID: 28322295 PMC5359573

[ref33] Liang W. Yang Y. Wang H. Wang H. Yu X. Lu Y. . (2019). Gut microbiota shifts in patients with gastric cancer in perioperative period. Medicine 98:e16626. doi: 10.1097/MD.0000000000016626, PMID: 31464899 PMC6736490

[ref34] Liberati A. Altman D. G. Tetzlaff J. Mulrow C. Gøtzsche P. C. Ioannidis J. P. A. . (2009). The PRISMA statement for reporting systematic reviews and meta-analyses of studies that evaluate health care interventions: explanation and elaboration. PLoS Med. 6:e1000100. doi: 10.1371/journal.pmed.1000100, PMID: 19621070 PMC2707010

[ref35] Lim M. Y. Hong S. Bang S. J. Chung W. H. Shin J. H. Kim J. H. . (2021). Gut microbiome structure and association with host factors in a Korean population. mSystems 6:e0017921. doi: 10.1128/mSystems.00179-21, PMID: 34342532 PMC8407462

[ref36] Liu C. Ng S. K. Ding Y. Lin Y. Liu W. Wong S. H. . (2022). Meta-analysis of mucosal microbiota reveals universal microbial signatures and dysbiosis in gastric carcinogenesis. Oncogene 41, 3599–3610. doi: 10.1038/s41388-022-02377-9, PMID: 35680985 PMC9270228

[ref37] Lo C. H. Kwon S. Wang L. Polychronidis G. Knudsen M. D. Zhong R. . (2021). Periodontal disease, tooth loss, and risk of oesophageal and gastric adenocarcinoma: a prospective study. Gut 70, 620–621. doi: 10.1136/gutjnl-2020-321949, PMID: 32690603 PMC7855151

[ref38] Lozupone C. A. Knight R. (2008). Species divergence and the measurement of microbial diversity. FEMS Microbiol. Rev. 32, 557–578. doi: 10.1111/j.1574-6976.2008.00111.x, PMID: 18435746 PMC2443784

[ref39] Mavridis D. Salanti G. (2014). How to assess publication bias: funnel plot, trim-and-fill method and selection models. Evid. Based Ment. Health 17:30. doi: 10.1136/eb-2013-101699, PMID: 24477535 PMC13404685

[ref40] Miao Y. Tang H. Zhai Q. Liu L. Xia L. Wu W. . (2022). Gut microbiota dysbiosis in the development and progression of gastric cancer. J. Oncol. 2022, 1–15. doi: 10.1155/2022/9971619 PMC9441395 36072968

[ref41] Nardone G. Compare D. (2015). The human gastric microbiota: is it time to rethink the pathogenesis of stomach diseases? United European Gastroenterol J 3, 255–260. doi: 10.1177/2050640614566846, PMID: 26137299 PMC4480535

[ref42] Nearing J. T. Douglas G. M. Hayes M. G. MacDonald J. Desai D. K. Allward N. . (2022). Microbiome differential abundance methods produce different results across 38 datasets. Nat. Commun. 13:342. doi: 10.1038/s41467-022-28034-z, PMID: 35039521 PMC8763921

[ref43] Nikitina D. Lehr K. Vilchez-Vargas R. Jonaitis L. V. Urba M. Kupcinskas J. . (2023). Comparison of genomic and transcriptional microbiome analysis in gastric cancer patients and healthy individuals. World J. Gastroenterol. 29, 1202–1218. doi: 10.3748/wjg.v29.i7.1202, PMID: 36926663 PMC10011954

[ref44] Oriuchi M. Lee S. Uno K. Sudo K. Kusano K. Asano N. . (2024). *Porphyromonas gingivalis* lipopolysaccharide damages mucosal barrier to promote gastritis-associated carcinogenesis. Dig. Dis. Sci. 69, 95–111. doi: 10.1007/s10620-023-08142-6, PMID: 37943385

[ref45] Park J. Y. Seo H. Kang C. S. Shin T. S. Kim J. W. Park J. M. . (2022). Dysbiotic change in gastric microbiome and its functional implication in gastric carcinogenesis. Sci. Rep. 12:4285. doi: 10.1038/s41598-022-08288-9, PMID: 35277583 PMC8917121

[ref46] Peng X. Yao S. Huang J. Zhao Y. Chen H. Chen L. . (2023). Alterations in bacterial community dynamics from noncancerous to gastric cancer. Front. Microbiol. 14:1138928. doi: 10.3389/fmicb.2023.1138928, PMID: 36970687 PMC10034189

[ref47] Pimentel-Nunes P. Barros A. Pita I. Miranda I. Conceição G. Borges-Canha M. . (2021). Gastric microbiome profile throughout gastric carcinogenesis: beyond helicobacter. Scand. J. Gastroenterol. 56, 708–716. doi: 10.1080/00365521.2021.1902560, PMID: 33915074

[ref48] Plottel C. S. Blaser M. J. (2011). Microbiome and malignancy. Cell Host Microbe 10, 324–335. doi: 10.1016/j.chom.2011.10.003, PMID: 22018233 PMC3264051

[ref49] Qi Y. F. Sun J. N. Ren L. F. Cao X. L. Dong J. H. Tao K. . (2019). Intestinal microbiota is altered in patients with gastric cancer from Shanxi province, China. Dig. Dis. Sci. 64, 1193–1203. doi: 10.1007/s10620-018-5411-y 30535886

[ref50] Rawla P. Barsouk A. (2019). Epidemiology of gastric cancer: global trends, risk factors and prevention. Prz. Gastroenterol. 14, 26–38. doi: 10.5114/pg.2018.80001, PMID: 30944675 PMC6444111

[ref51] Sohn S. H. Kim N. Jo H. J. Kim J. Park J. H. Nam R. H. . (2017). Analysis of gastric body microbiota by pyrosequencing: possible role of bacteria other than *Helicobacter pylori* in the gastric carcinogenesis. Journal Cancer Prevent. 22, 115–125. doi: 10.15430/JCP.2017.22.2.115, PMID: 28698866 PMC5503224

[ref52] Spiegelhauer M. R. Kupcinskas J. Johannesen T. B. Urba M. Skieceviciene J. Jonaitis L. . (2020). Transient and persistent gastric microbiome: adherence of bacteria in gastric cancer and dyspeptic patient biopsies after washing. J. Clin. Med. 9:1882. doi: 10.3390/jcm9061882, PMID: 32560179 PMC7357088

[ref53] Sun Q. H. Zhang J. Shi Y. Y. Zhang J. Fu W. W. Ding S. G. (2022). Microbiome changes in the gastric mucosa and gastric juice in different histological stages of *Helicobacter pylori*-negative gastric cancers. World J. Gastroenterol. 28, 365–380. doi: 10.3748/wjg.v28.i3.365, PMID: 35110955 PMC8771614

[ref54] Sung H. Ferlay J. Siegel R. L. Laversanne M. Soerjomataram I. Jemal A. . (2021). Global cancer statistics 2020: GLOBOCAN estimates of incidence and mortality worldwide for 36 cancers in 185 countries. CA Cancer J. Clin. 71, 209–249. doi: 10.3322/caac.21660, PMID: 33538338

[ref55] Taha S. F. M. Bhassu S. Omar H. Raju C. S. Rajamanikam A. Govind S. K. P. . (2023). Gut microbiota of healthy Asians and their discriminative features revealed by metagenomics approach. 3 Biotech 13:275. doi: 10.1007/s13205-023-03671-3 PMC10338424 37457869

[ref56] Tang Q. Jin G. Wang G. Liu T. Liu X. Wang B. . (2020). Current sampling methods for gut microbiota: a call for more precise devices. Front. Cell. Infect. Microbiol. 10:151. doi: 10.3389/fcimb.2020.00151, PMID: 32328469 PMC7161087

[ref57] Tropini C. Moss E. L. Merrill B. D. Ng K. M. Higginbottom S. K. Casavant E. P. . (2018). Transient osmotic perturbation causes long-term alteration to the gut microbiota. Cell 173, 1742–54.e17. doi: 10.1016/j.cell.2018.05.008, PMID: 29906449 PMC6061967

[ref58] Wang Z. Gao X. Zeng R. Wu Q. Sun H. Wu W. . (2020). Changes of the gastric mucosal microbiome associated with histological stages of gastric carcinogenesis. Front. Microbiol. 11:997. doi: 10.3389/fmicb.2020.00997, PMID: 32547510 PMC7272699

[ref59] Wang L. L. Liu J. X. Yu X. J. Si J. L. Zhai Y. X. Dong Q. J. (2018). Microbial community reshaped in gastric cancer. Eur. Rev. Med. Pharmacol. Sci. 22, 7257–7264. doi: 10.26355/eurrev_201811_16260, PMID: 30468469

[ref60] Wang L. Zhou J. Xin Y. Geng C. Tian Z. Yu X. . (2016). Bacterial overgrowth and diversification of microbiota in gastric cancer. Eur. J. Gastroenterol. Hepatol. 28, 261–266. doi: 10.1097/MEG.0000000000000542, PMID: 26657453 PMC4739309

[ref61] Wei Q. Zhang Q. Wu Y. Han S. Yin L. Zhang J. . (2023). Analysis of bacterial diversity and community structure in gastric juice of patients with advanced gastric cancer. Discov. Oncol. 14:7. doi: 10.1007/s12672-023-00612-7, PMID: 36662326 PMC9860007

[ref62] Wells G. Shea B. J. O'Connell D. Peterson J. Welch V. Losos M. . (2000). The Newcastle-Ottawa scale (NOS) for assessing the quality of nonrandomized studies in meta-analyses. (2000). Available at: https://api.semanticscholar.org/CorpusID:79550924.

[ref63] Widder S. Allen R. J. Pfeiffer T. Curtis T. P. Wiuf C. Sloan W. T. . (2016). Challenges in microbial ecology: building predictive understanding of community function and dynamics. ISME J. 10, 2557–2568. doi: 10.1038/ismej.2016.45, PMID: 27022995 PMC5113837

[ref64] World Health Organization. Global health estimates 2020: Deaths by cause, age, sex, by country and by region, 2000–2019 (2020). Available at: http://who.int/data/gho/data/themes/mortality-and-global-health-estimates/ghe-leading-causes-of-death.

[ref65] Wu Z. F. Zou K. Wu G. N. Jin Z. J. Xiang C. J. Xu S. . (2021). A comparison of tumor-associated and non-tumor-associated gastric microbiota in gastric cancer patients. Dig. Dis. Sci. 66, 1673–1682. doi: 10.1007/s10620-020-06415-y, PMID: 32591968

[ref66] Yang P. Zhang X. Xu R. Adeel K. Lu X. Chen M. . (2022). Fungal microbiota dysbiosis and ecological alterations in gastric cancer. Front. Microbiol. 13:889694. doi: 10.3389/fmicb.2022.889694, PMID: 35572666 PMC9100745

[ref67] Yu G. Torres J. Hu N. Medrano-Guzman R. Herrera-Goepfert R. Humphrys M. S. . (2017). Molecular characterization of the human stomach microbiota in gastric cancer patients. Front. Cell. Infect. Microbiol. 7:302. doi: 10.3389/fcimb.2017.00302, PMID: 28730144 PMC5498480

[ref68] Zhang X. Li C. Cao W. Zhang Z. (2021). Alterations of gastric microbiota in gastric cancer and precancerous stages. Front. Cell. Infect. Microbiol. 11:559148. doi: 10.3389/fcimb.2021.559148, PMID: 33747975 PMC7966516

