# Corrigendum: Gut microbiome alterations during gastric cancer: evidence assessment of case–control studies

**DOI:** 10.3389/fmicb.2024.1448265

**Published:** 2024-06-25

**Authors:** Ruimin Zhang, Yingxin Wu, Wantao Ju, Senlin Wang, Yanjun Liu, Hongmei Zhu

**Affiliations:** ^1^Institute of Biomedical Engineering, College of Medicine, Southwest Jiaotong University, Chengdu, China; ^2^Section for Gastrointestinal Surgery, Department of General Surgery, The Third People's Hospital of Chengdu, Affiliated Hospital of Southwest Jiaotong University & The Second Affiliated Hospital of Chengdu, Chongqing Medical University, Chengdu, China; ^3^School of Life Science and Engineering, Southwest Jiaotong University, Chengdu, China; ^4^Medical Research Center, The Third People's Hospital of Chengdu, The Affiliated Hospital of Southwest Jiaotong University, Chengdu, China

**Keywords:** gastric cancer, gastric microbiota, stomach, carcinogenesis, meta-analysis

In the published article, there was an error in Yingxin Wu, Wantao Ju and Hongmei Zhu's affiliation. Yingxin Wu, Wantao Ju and Hongmei Zhu were not affiliated with affiliation 1, “Institute of Biomedical Engineering, College of Medicine, Southwest Jiaotong University, Chengdu, China” and this has been removed from their affiliations. The correct author list appears below.

Ruimin Zhang^1, 2^^†^, Yingxin Wu^2^^†^, Wantao Ju^2, 3^, Senlin Wang^1, 2^, Yanjun Liu^1, 2*^ and Hongmei Zhu^2, 4*^

The authors apologize for this error and state that this does not change the scientific conclusions of the article in any way. The original article has been updated.

