# Supplementary material for: Gut microbiome alterations during gastric cancer: evidence assessment of case–control studies
Source: Front Microbiol. 2024 May 15;15:1406526. doi: 10.3389/fmicb.2024.1406526 (PMC11133546; doi:10.3389/fmicb.2024.1406526)
Supplement: Supplementary file 1 [file Table_1.DOCX]

**Supporting Material: Gut Microbiome Alterations during Gastric Cancer: Evidence Assessment of Case-control Studies**

**Table S1: Search Strategy**

**Table S2: Quality assessment of the included studies Newcastle-Ottawa Scale for assessing the quality of studies in meta-analysis**

**Table S3: Description of methods and results of microbial diversity and richness assessment.**

**Table S4: Differences in the relative abundance of bacteria at phylum level.**

**Table S5: Significant differences in the relative abundance of bacteria at genus level between gastric cancer and non-gastric cancer patients.**

**Table S6: Meta regression analysis of the relationship between outcomes and characteristics of studies.**

**Table S7: Microbial community structure sensitivity analysis excluding small samples.**

**Table S8: Publication Bias Assessment Results for Microbial Diversity and Abundance in Studies.**

**Figure S1: Forest plot of the differences in alpha diversity by Shannon index.**

**Table S1. Search Strategy**

| **Database** | **Search strategy** |
| --- | --- |
| PubMed | (microbio*[Title/Abstract]) AND ("stomach neoplasms"[MeSH Terms] OR "cancer of stomach"[Title/Abstract] OR "stomach cancers"[Title/Abstract] OR "gastric cancer*"[Title/Abstract]) |
| Web of Science | TS=(Stomach Neoplasms) AND TI=(microbio*) |
| EMBASE | ('stomach cancer'/exp OR 'stomach tumor'/exp) AND microbio*:ti,ab,kw |
| Cochrane | (MeSH descriptor: [Stomach Neoplasms] explode all trees OR (cancer of stomach):ti,ab,kw OR (stomach cancers):ti,ab,kw OR (gastric cancer*):ti,ab,kw) AND (microbio*):ti,ab,kw |

Systematic search was performed on April 4, 2023.

**Table S2. Quality Assessment of The Included Studies Newcastle-Ottawa Scale for Assessing The Quality of Studies in Meta-analysis.**

| Study ID | Selection | | | | Comparability Control for important facter a | Exposure | | | Scores |
| --- | --- | --- | --- | --- | --- | --- | --- | --- | --- |
|  | **Adequate definition of cases** | **Representativeness of the cases** | **Selection of controls** | **Definition of controls** |  | **Ascertainment of exposure** | **Same method of ascertainment for cases and controls** | **No response**  **rate** |  |
| Eun,2014 | 1 | 1 | 0 | 1 | 2 | 1 | 1 | 1 | 8 |
| Jo,2016 | 1 | 1 | 1 | 1 | 1 | 1 | 1 | 1 | 8 |
| Wang,2016 | 1 | 1 | 0 | 1 | 1 | 1 | 1 | 1 | 7 |
| Castaño,2017 | 1 | 1 | 0 | 1 | 0 | 1 | 1 | 1 | 6 |
| Li,2017 | 1 | 1 | 1 | 1 | 1 | 1 | 1 | 1 | 8 |
| Sohn, 2017 | 0 | 1 | 0 | 1 | 1 | 1 | 1 | 1 | 6 |
| Yu,2017 | 1 | 1 | 0 | 1 | 2 | 1 | 1 | 1 | 8 |
| Coker,2018 | 1 | 1 | 0 | 1 | 1 | 1 | 1 | 1 | 7 |
| Ferreira,2018 | 1 | 1 | 0 | 1 | 1 | 1 | 1 | 1 | 7 |
| Hsieh,2018 | 1 | 1 | 0 | 1 | 1 | 1 | 1 | 1 | 7 |
| Chen,2019 | 0 | 1 | 0 | 1 | 0 | 1 | 1 | 1 | 5 |
| Gunathilake,2019 | 1 | 1 | 0 | 1 | 1 | 1 | 1 | 1 | 7 |
| Liang,2019 | 1 | 1 | 1 | 1 | 1 | 1 | 1 | 1 | 8 |
| Qi,2019 | 1 | 1 | 1 | 1 | 2 | 1 | 1 | 1 | 9 |
| Gantuya,2020 | 1 | 1 | 1 | 1 | 0 | 1 | 1 | 1 | 7 |
| Gunathilake,2020 | 1 | 1 | 1 | 1 | 2 | 1 | 1 | 1 | 9 |
| Wang,2020 | 1 | 1 | 1 | 1 | 0 | 1 | 1 | 1 | 7 |
| Deng,2021 | 1 | 1 | 1 | 1 | 2 | 1 | 1 | 1 | 9 |
| Gunathilake,2021 | 1 | 1 | 0 | 1 | 2 | 1 | 1 | 1 | 8 |
| Kadeerhan,2021 | 1 | 1 | 1 | 1 | 0 | 1 | 1 | 1 | 7 |
| Pimentel-Nunes,2021 | 1 | 1 | 0 | 1 | 1 | 1 | 1 | 1 | 7 |
| Wu,2021 | 1 | 1 | 0 | 1 | 1 | 1 | 1 | 1 | 7 |
| Zhang,2021 | 0 | 1 | 1 | 1 | 2 | 1 | 1 | 1 | 8 |
| He,2022 | 1 | 1 | 1 | 1 | 0 | 1 | 1 | 1 | 7 |
| Kim,2022 | 1 | 1 | 0 | 1 | 1 | 1 | 1 | 1 | 7 |
| LI,2022 | 1 | 0 | 0 | 1 | 1 | 1 | 1 | 1 | 6 |
| Miao,2022 | 1 | 1 | 0 | 1 | 1 | 1 | 1 | 1 | 7 |
| Park,2022 | 1 | 1 | 1 | 1 | 1 | 1 | 1 | 1 | 8 |
| Sun,2022 | 1 | 1 | 0 | 1 | 1 | 1 | 1 | 1 | 7 |
| Yang,2022 | 1 | 1 | 0 | 1 | 0 | 1 | 1 | 1 | 6 |
| Nikitina,2023 | 1 | 1 | 1 | 1 | 1 | 1 | 1 | 1 | 8 |
| Peng,2023 | 1 | 1 | 1 | 1 | 1 | 1 | 1 | 1 | 8 |
| Wei,2023 | 1 | 1 | 1 | 1 | 0 | 1 | 1 | 1 | 7 |

**Table S3.** **Description of methods and results of microbial diversity and richness assessment.**

| Study ID | Indicators of α Diversity | Result | Indicators of β Diversity | Result |
| --- | --- | --- | --- | --- |
| Eun,2014 | Shannon | ↑ | - | - |
| Jo,2016 | NA | no significant differences | - | - |
| Wang,2016 | Chao1 | ↑ | - | - |
| Castaño,2017 | Species richness, phylogenetic diversity | ↑ | - | - |
| Li,2017 | Shannon, phylogenetic diversity | ↓ | - | - |
| Sohn, 2017 | - | - | - | - |
| Yu,2017 | NA | Chinese: significant differences | - | - |
| Coker,2018 | chao1 ^1^ | ↓ | NA | significant differences |
| Ferreira,2018 | Shannon | ↓ | NA | significant differences |
| Hsieh,2018 | - | - | - | - |
| Chen,2019 | Shannon, PD whole tree, Chao 1, ACE | ↑ | ANOSIM, NMDS | significant differences |
| Gunathilake,2019 | Shannon | ↓ | ANOSIM, PCoA | significant differences |
| Liang,2019 | Simpson, Chao 1, ACE, Shannon | Simpson, Chao 1, ACE ↑  Shannon ↓ | - | - |
| Qi,2019 | Sobs | ↑ | ANOSIM, PCoA | significant differences |
| Gantuya,2020 | Shannon | ↓ | - | - |
| Gunathilake,2020 | Species richness, Shannon | Species richness ↑  Shannon ↓ | ANOSIM, PCoA | significant differences |
| Wang,2020 | NA | ↓ | NMDS, PCoA | significant differences |
| Deng,2021 | Shannon | ↓ | NA | revealed an obvious separation |
| Gunathilake,2021 | Species richness, Shannon, Pilouevenness | Species richness ↑，Shannon, Pilouevenness ↓ | - | - |
| Kadeerhan,2021^3^ | Species richness, Shannon | ↑ | NA | significant differences |
| Pimentel-Nunes,2021 | Microbial diversity, richness | ↑ | - | - |
| Wu,2021 | coverage, Simpson, Ace, Chao, observed OTUs, Shannon | coverage, Simpson ↑  Ace, Chao, observed OTUs, Shannon ↓ | PCoA | significant differences |
| Zhang,2021 | Chao 1 | ↓ | - | - |
| He,2022 | Microbial diversity, species richness | ↑ | - | - |
| Kim,2022 | Richness | ↓ | NA | significant differences |
| LI,2022 | Chao 1, Shannon | ↑ | PCoA | significant differences |
| Miao,2022 | Simpson, Shannon ^2^ | Simpson ↑ Shannon ^2^ ↓ | NA | significant differences |
| Park,2022 | Shannon, Simpson, Chao 1, Observed | ↓ | NA | significant differences |
| Sun,2022 | Simpson, Chao 1, OUTs | ↓ | NA | ↓ |
| Yang,2022 | Chao1, observed species, PD Whole tree | ↓ | NA | significant differences |
| Nikitina,2023 | - | - | NA | significant differences |
| Peng,2023 | Richness,Simpson, Shannon | ↓ | NA | significant differences |
| Wei,2023 | NA | significant differences | NA | significant differences |

↑: Increase; ↓: Decrease; NA: Not available; ^1^: IM, GC VS SG; 2: SG VS GC; 3: CAG, IM, DYS/GC VS normal/SG and comparing DYS/GC VS IM; PD: phylogenetic distance; OTUs: operational taxonomic units; PCoA: Principal Coordinates analysis; ACE: Abundance-based Coverage Estimator; ANOSIM: Analysis of Similarities; NMDS: Non-metric Multidimensional Scaling; IM: Intestinal metaplasia; GC: Gastric cancer; SG: Superficial gastritis; CAG: Chronic atrophic gastritis; DYS: Dysplasia.

**Table S4: Differences in the relative abundance of bacteria at phylum level.**

| Study ID | *Actinobacteria* | *Bacteroidetes* | | *Firmicutes* | | *Fusobacteria* | | *Proteobacteria* | | Other | |
| --- | --- | --- | --- | --- | --- | --- | --- | --- | --- | --- | --- |
| Eun,2014 | - | - | | - | | - | | - | | *Epsilonproteobacteria ↓* | |
| Jo,2016 | ↑ | - | | - | | - | | ↓ | | - | |
| Wang,2016 | - | - | | - | | - | | - | | - | |
| Castaño,2017 | - | - | | - | | - | | - | | - | |
| Li,2017 | - | ↑ | | - | | - | | ↑ | | - | |
| Sohn, 2017 | ↑ | - | | ↑ | | - | | ↑ | | - | |
| Yu,2017 | - | ↑ ^1^ | | ↑ ^1^ | | ↑ ^1^ | | ↑ ^1^ | | *Spirochaetes*↑ ^1^ | |
| Coker,2018 | - | - | | - | | ↑ | | - | | - | |
| Ferreira,2018 | - | - | | - | | - | | - | | - | |
| Hsieh,2018 | - | - | | - | | - | | - | | - | |
| Chen,2019 | ↑ | ↑ | | ↑ | | ↑ | | ↓ | | *Acidobacteria*↑ | |
| Gunathilake,2019 | - | - | | - | | - | | - | | - | |
| Liang,2019 | - | ↓ | | - | | - | | ↑ | | - | |
| Qi,2019 | ↑ | - | | ↓ | | - | | ↑ | | - | |
| Gantuya,2020 | - | - | | ↑ | | - | | - | | - | |
| Gunathilake,2020 | - | - | | - | | - | | - | | - | |
| Wang,2020 | ↑ | ↑ | | ↑ | | ↑ | | ↑ | | *Acidobacteria* ↑ *; Verrucomicrobia* ↓ | |
| Deng,2021 | ↓ | - | | - | | - | | ↑ | | - | |
| Gunathilake,2021 | ↓ | | ↓ | | ↓ | | ↓ | | ↓ | | - |
| Kadeerhan,2021 | ↑ | | ↑ | | ↑ | | - | | ↓ | | - |
| PimentelNunes,2021 | - | | ↓ | | ↑ | | - | | ↓ | | - |
| Wu,2021 | - | | - | | - | | - | |  | | - |
| Zhang,2021 | - | | - | | - | | - | | - | | - |
| He,2022 | - | | - | | ↑ | | - | | ↓ | | - |
| Kim,2022 | - | | - | | - | | - | | - | | - |
| LI,2022 | ↓ | | ↑ | | ↓ | | - | | ↑ | | - |
| Miao,2022 | - | | - | | - | | - | | - | | - |
| Park,2022 | ↓ ^2^ | | - | | - | | - | | - | | *Verrucomicrobia, Defrribacteres* ↑ |
| Sun,2022 | ↓ | | - | | ↑ | | ↑ | | ↓ | | - |
| Yang,2022 | - | | - | | - | | - | | - | | *Basidiomvcota* ↑*; Rozelloycota* ↓ |
| Nikitina,2023 | - | | - | | - | | - | | - | | - |
| Peng,2023 | - | | - | | ↑ | | - | | ↓ | | *Spirochaetota* ↓ |
| Wei,2023 | - | | - | | - | | - | | - | | - |

^1^:In China; ^2^: AGC VS GA; ↑: Increase; ↓: Decrease; AGC: Advanced gastric cancer; GA: Gastritis.

**Table S5: Significant differences in the relative abundance of bacteria at genus level between gastric cancer and non-gastric cancer patients.**

| Genus | Significantly Higher in Gastric Cancer | | Significantly Lower in Gastric Cancer | |
| --- | --- | --- | --- | --- |
|  | **n** | **[Study]** | **n** | **[Study]** |
| *Achromobacter* | 2 | Li TH, 2017; Ferreira RM, 2018 | - | - |
| *Acinetobacter* | 3 | Chen XH, 2019; Kadeerhan G, 2021; Park JY, 2022 | 2 | Coker OO, 2018; Wang Z, 2020 |
| *Bacillus* | 2 | Chen XH, 2019; Kadeerhan G, 2021 | - | - |
| *Bacteroides* | 3 | Chen XH, 2019; Gunathilake M, 2021; He F, 2022 | 2 | Liang W, 2019; Qi YF, 2019 |
| *Bifidobacterium* | 1 | Sun QH, 2022 | 2 | He F, 2022; LI N, 2022 |
| *Campylobacter* | 1 | Kadeerhan G, 2021 | 3 | Wu ZF, 2021; Kim22; Peng X, 2023 |
| *Capnocytophaga* | 2 | Kadeerhan G, 2021; Zhang X, 2021 | - | - |
| *Clostridium* | 3 | Ferreira RM, 2018; Hsieh YY, 2018; Liang W, 2019 | - | - |
| *Dialister* | 2 | Coker OO, 2018; Wu ZF, 2021 | - | - |
| *Enterococcus* | 3 | Gantuya B, 2020; Pimentel- Nunes P, 2021; Miao Y, 2022 | 1 | He F, 2022 |
| *Escherichia* | 4 | Liang W, 2019; Qi YF, 2019; Gantuya B, 2020;  LI N, 2022 | 1 | Peng X, 2023 |
| *Faecalibacterium* | 1 | He F, 2022 | 2 | Qi YF, 2019; LI N, 2022 |
| *Fusobacterium* | 4 | Coker OO, 2018; Hsieh YY, 2018; Chen XH, 2019; Gantuya B, 2020 | 2 | Wang Z, 2020; Wu ZF, 2021 |
| *Gemella* | 3 | Coker OO, 2018; Pimentel- Nunes P, 2021; Wei Q, 2023 | 1 | Wang Z, 2020 |
| *Helicobacter* | 5 | Gunathilake MN, 2019; Wang Z, 2020; Wu ZF, 2021; Park JY, 2022; Wei Q, 2023 | 6 | Yu17; Ferreira RM, 2018; Hsieh YY, 2018; Chen XH, 2019 Gantuya B, 2020; Kadeerhan G, 2021 |
| *Klebsiella* | 2 | LI N, 2022; Qi YF, 2019 | - | - |
| *Lactobacillus* | 10 | Eun CS, 2014; Castaño-Rodríguez N, 2017; Ferreira RM, 2018; Hsieh YY, 2018; Gantuya B, 2020; Wang Z, 2020; Wu ZF, 2021; Sun QH, 2022; Peng X, 2023; Wei Q, 2023 | 2 | Chen XH, 2019; Park JY, 2022 |
| *Lactococcus* | 1 | Castaño-Rodríguez N, 2017 | 1 | Gunathilake MN, 2019 |
| *Neisseria* | 1 | Kadeerhan G, 2021 | 4 | Ferreira RM, 2018; Pimentel- Nunes P, 2021; Wu ZF, 2021; Peng X, 2023 |
| *Nitrospirae* | 1 | Wang L, 2016 | 1 | Wang Z, 2020 |
| *Peptostreptococcus* | 4 | Coker OO, 2018; Chen XH, 2019; Gantuya B, 2020; Kadeerhan G, 2021 | 1 | Gunathilake M, 2021 |
| *Phyllobacterium* | 1 | Ferreira RM, 2018 | 1 | Wu ZF, 2021 |
| *Porphyromonas* | - | - | 2 | Wang Z, 2020; Wu ZF, 2021 |
| *Prevotella* | 4 | Chen XH, 2019; Gunathilake MN, 2019; LI N, 2022; Park JY, 2022 | 4 | Ferreira RM, 2018; Gunathilake M, 2021; Wu ZF, 2021; Sun QH, 2022 |
| *Pseudomonas* | 3 | LI N, 2022; Kadeerhan G, 2021; Nikitina D, 2023 | 1 | Wang Z, 2020 |
| *Rhodococcus* | 2 | Ferreira RM, 2018; Wu ZF, 2021 | 2 | Sun QH, 2022; Wei Q, 2023 |
| *Roseburia* | 1 | He F, 2022 | 2 | LI N, 2022; Miao Y, 2022 |
| *Rothia* | - | - | 2 | Wang Z, 2020; Wu ZF, 2021 |
| *Slackia* | 2 | Coker OO, 2018; Zhang X, 2021 | - | - |
| *Sphingomonas* | 2 | Chen XH, 2019; Kadeerhan G, 2021 | 1 | Wang Z, 2020 |
| *Streptococcus* | 9 | Eun CS, 2014; Sohn SH, 2017; Chen XH, 2019; Gantuya B, 2020; Pimentel- Nunes P, 2021; He F, 2022; Sun QH, 2022; Peng X, 2023; Wei Q, 2023 | 2 | Ferreira RM, 2018; Wu ZF, 2021 |
| *Veillonella* | 3 | Liang W, 2019; Park JY, 2022; Sun QH, 2022 | 1 | Wu ZF, 2021 |

**Table S6: Meta regression analysis of the relationship between outcomes and characteristics of studies.**

| **Outcome** | **Studies (n)** | **Factor** | **Coefficient** | **z** | ***p* Value** |
| --- | --- | --- | --- | --- | --- |
| ***Actinobacteria*** | 10 | Method | -20.59842 | -2.630 | 0.009 |
|  |  | Database | 18.17374 | 2.650 | 0.008 |
| ***Bacteroidetes*** | 9 | country | -21.91816 | -7.090 | 0.000 |
| ***Firmicutes*** | 12 | country | 9.307176 | 2.360 | 0.018 |
|  |  | Sample source | -19.25102 | -2.730 | 0.006 |

| Table S7: Microbial community structure sensitivity analysis excluding small samples. | | | | | | | | | |
| --- | --- | --- | --- | --- | --- | --- | --- | --- | --- |
| Outcome Measure | | **No. of studies** | **Pre-exclusion** | | | **No. of Studies Excluded** | **Post-exclusion** | | |
|  |  |  | **Log Odds Ratio (95% CI)** | ***p*** | **I^2^** |  | **Log Odds Ratio (95% CI)** | ***p*** | **I^2^** |
| α diversity | Chao1 | 8 | 1.015 (-5.682, 7.713) | 0.766 | 95.70% | 4 | 5.730 (-5.077, 16.538) | 0.299 | 96.70% |
|  | Shannon | 17 | -5.078 (-9.470, -0.686) | 0.023 | 95.30% | 4 | -5.461 (-10.873, -0.049) | 0.048 | 95.90% |
|  | Species richness | 8 | 5.100 (-1.444, 11.643) | 0.127 | 95.50% | 1 | 4.812 (-2.589, 12.213) | 0.203 | 95.90% |
|  | Simpson | 5 | 1.067 (-6.936, 9.069) | 0.794 | 95.10% | 2 | -3.323 (-14.572,7.926) | 0.563 | 95.90% |
| Phylum | *Actinobacteria* | 10 | -0.533 (-6.701, 5.636) | 0.866 | 95.90% | 2 | -0.428 (-7.860, 7.057) | 0.916 | 96.50% |
|  | *Bacteroidetes* | 9 | 3.049 (-3.852, 9.950) | 0.386 | 96.40% | 1 | 4.370 (-3.182, 11.922) | 0.257 | 96.60% |
|  | *Firmicutes* | 13 | 1.400 (-4.148, 6.947) | 0.621 | 96.10% | 1 | 1.092 (-4.855, 7.039) | 0.719 | 96.30% |
|  | *Proteobacteria* | 16 | -1.479 (-6.075, 3.118) | 0.528 | 95.40% | 2 | -2.59 (-7.657, 2.476) | 0.316 | 95.70% |
| Genus | *Bacteroides* | 5 | 2.476 (-7.024, 11.975) | 0.609 | 96.60% | 1 | 4.974 (-6.189,16.136) | 0.383 | 96.90% |
|  | *Campylobacter* | 4 | -2.617 (-15.014, 9.780) | 0.679 | 96.60% | 2 | -0.033 (-19.049, 18.983) | 0.997 | 97.90% |
|  | *Clostridium* | 3 | 7.994 (5.706, 10.283) | 7.55E-12 | 0.00% | 1 | 8.227 (5.137, 11.317) | 1.80E-07 | 17.70% |
|  | *Fusobacterium* | 6 | 3.257 (-4.420, 10.934) | 0.406 | 95.60% | 2 | 5.173 (-5.237, 15.583) | 0.330 | 96.40% |
|  | *Helicobacter* | 11 | -1.138 (-7.205,4.930) | 0.713 | 96.20% | 2 | -3.055 (-10.124, 4.013) | 0.397 | 96.60% |
|  | *Lactobacillus* | 12 | 5.325 (0.470, 10.181) | 0.032 | 94.00% | 5 | 4.383 (-2.746, 11.512) | 0.228 | 95.60% |
|  | *Neisseria* | 5 | -4.685 (-12.579, 3.208) | 0.245 | 95.00% | 2 | -2.780 (-15.323, 9.762) | 0.664 | 96.70% |
|  | *Streptococcus* | 12 | 6.383 (1.548, 11.218) | 0.010 | 93.30% | 4 | 6.462 (-0.203, 13.128) | 0.057 | 95.00% |
|  | *Prevotella* | 8 | 0.220 (-7.313, 7.754) | 0.954 | 96.60% | 2 | 0.390 (-9.163, 9.943) | 0.936 | 97.20% |
|  | *Pseudomonas*  *Achromobacter*  *Bacillus*  *Capnocytophaga*  *Dialister*  *Klebsiella*  *Slackia*  *Porphyromonas*  *Rothia* | 4  2  2  2  2  2  2  2  2 | 4.248 (-5.515, 14.010)  8.716 (5.923, 11.510)  9.661 (6.876, 12.446)  8.643 (5.847, 11.439)  8.995 (6.204, 11.787)  9.141 (6.203, 12.080)  8.909 (6.116,11.703)  -8.602 (-11.396, -5.808)  -8.602 (-11.396, -5.808) | 0.394  2.00E-09  1.06E-10  2.00E-09  1.35E-09  2.00E-09  1.36E-09  2.00E-09  2.00E-09 | 95.90%  0.00%  0.00%  0.00%  0.00%  0.00%  0.00%  0.00%  0.00% | 1  0  0  0  0  0  0  0  0 | 3.121 (-9.639, 15.881)  8.716 (5.923, 11.510)  9.661 (6.876, 12.446)  8.643 (5.847, 11.439)  8.995 (6.204, 11.787)  9.141 (6.203, 12.080)  8.909 (6.116,11.703)  -8.602 (-11.396, -5.808)  -8.602 (-11.396, -5.808) | 0.632  2.00E-09  1.06E-10  2.00E-09  1.35E-09  2.00E-09  1.36E-09  2.00E-09  2.00E-09 | 96.80%  0.00%  0.00%  0.00%  0.00%  0.00%  0.00%  0.00%  0.00% |

**Table S8: Publication Bias Assessment Results for Microbial Diversity and Abundance in Studies.**

| **Outcome** | **No. of studies** | **Egger's test** | | **Begg's test** | | **Trim-and-fill** | | | |
| --- | --- | --- | --- | --- | --- | --- | --- | --- | --- |
|  |  | **t** | ***p*** | **Z** | ***p*** | **No. of studies added** | **Location** | **Adjusted result** | ***p*** |
| **Shannon** | 17 | 0.58 | 0.567 | 2.88 | 0.004 | 0 | NA | 0.006(0.002,0.016) | 0.000 |
| ***Actinobacteria*** | 10 | 0.51 | 0.626 | 0.36 | 0.721 | 0 | NA | 0.568(0.163,1.983) | 0.375 |
| ***Firmicutes*** | 13 | -0.36 | 0.728 | 0.67 | 0.502 | 0 | NA | 3.514(1.220,10.120) | 0.020 |
| ***Proteobacteria*** | 16 | 0.72 | 0.485 | 1.04 | 0.300 | 3 | left | 0.014(0.006,0.034) | 0.000 |
| ***Helicobacter*** | 12 | 0.55 | 0.599 | 0.78 | 0.436 | 2 | left | 0.020(0.007,0.059) | 0.000 |
| ***Lactobacillus*** | 12 | 0.05 | 0.960 | 2.81 | 0.005 | 3 | left | 51.760(18.647,143.678) | 0.000 |
| ***Streptococcus*** | 11 | 0.01 | 0.992 | 2.18 | 0.029 | 2 | left | 52.871(17.631,158.546) | 0.000 |

**Figure S1.** Forest plot of the differences in alpha diversity by Shannon index.**
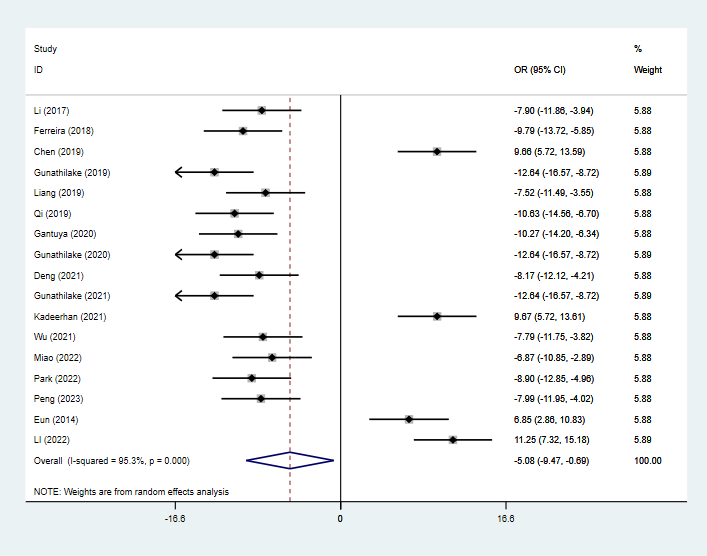
**
